# Atrazine Causes Autophagy- and Apoptosis-Related Neurodegenerative Effects in Dopaminergic Neurons in the Rat Nigrostriatal Dopaminergic System

**DOI:** 10.3390/ijms160613490

**Published:** 2015-06-12

**Authors:** Xiao-Yao Song, Jia-Nan Li, Yan-Ping Wu, Bo Zhang, Bai-Xiang Li

**Affiliations:** Department of Hygienic Toxicology, School of Public Health, Harbin Medical University, 157 Baojian Road, Nan Gang District, Harbin 150081, China; E-Mails: hedgehogviv@outlook.com (X.-Y.S.); love512133nan@163.com (J.-N.L.); Wuyanping_1964@aliyun.com (Y.-P.W.); sky_zhangbo_sea@163.com (B.Z.)

**Keywords:** atrazine, autophagy, apoptosis, neurotoxicity, wistar rats, dopaminergic neuron

## Abstract

Atrazine (2-chloro-4-ethytlamino-6-isopropylamine-1,3,5-triazine; ATR) is widely used as a broad-spectrum herbicide. Animal studies have demonstrated that ATR exposure can cause cell death in dopaminergic neurons. The molecular mechanisms underlying ATR-induced neuronal cell death, however, are unknown. In this study, we investigated the autophagy and apoptosis induced by ATR in dopaminergic neurons* in vivo*. Wistar rats were administered with ATR at doses of 10, 50 and 100 mg/kg body weight by oral gavage for three months. In terms of histopathology, the expression of autophagy- and apoptosis-related genes as well as proteins related to the Beclin-1/B-cell lymphoma 2 (Bcl-2) autophagy and apoptosis pathways were examined in the rat nigrostriatal dopaminergic system. We observed degenerative micromorphology indicative of neuronal apoptosis and mitochondrial autophagy by electron microscopy in ATR-exposed rat striatum. The rat ventral mesencephalon in the ATR-exposed groups also showed increased expression of Beclin-1, LC3-II, Bax and Caspase-9, and decreased expression of tyrosine hydroxylase (TH), Bcl-xl and Bcl-2. These findings indicate that ATR may induce autophagy- and apoptosis-related changes in doparminergic neurons. Furthermore, this induction may be regulated by the Beclin-1 and Bcl-2 autophagy and apoptosis pathways, and this may help to better understand the mechanism underlying the neurotoxicity of ATR.

## 1. Introduction

Atrazine (6-chloro-*N*-ethyl-*N′*-(1-methylethyl)-1,3,5-triazine-2,4-diamine, ATR) is the most commonly used broad-spectrum herbicide in agricultural crops such as corn, sorghum, and sugarcane [1,2]. Although farm usage of ATR is regulated in the EU, ATR remains one of the most commonly used pesticides in the world, and it is continually detected in the ground water in the US, Italy and Greece [3,4,5,6]. Several studies have demonstrated that ATR is an endocrine disruptor, and chronic ATR exposure results in high accumulative levels in multiple species, including frogs [7], fish [8] and rodents [9,10,11]. According to a report by the European Union, although residual ATR density decreased with increasing agricultural soil depth, it remained a significant environmental and biological hazard at the concentrations detected [12]. Due to their high and consistent ATR exposure, the health of farm workers and the residual metabolites that can be found in their urine is of particular concern [13]. Substantial levels of ATR and its metabolic products have been detected in urine samples from a farming community living within the proximity of herbicide use [14]. Additionally, an increased incidence of Parkinson’s disease (PD) in agricultural workers in rural environments has been attributed to ATR exposure [15,16].

Animal studies have indicated that ATR exposure reduces pituitary-testis hormone (luteotropic hormone, testosterone and follicle-stimulating hormone) levels, increases free triiodothyronine [17,18,19], and affects nervous system function. In addition, ATR induces ovarian functional changes via the regulation of hypothalamic catecholamines, especially norepinephrine (NE) and dopamine (DA) [20]. ATR exposure also decreased the level of striatal DA and reduced the number of tyrosine hydroxylase (TH)-positive dopaminergic neurons in both substantia nigra pars compacta (SNpc) and ventral tegmental area (VTA) in mice [21]. Furthermore, monoamine levels decreased in the dopaminergic systems of the brain after ATR exposure [22]. Decreased DA levels and dopaminergic neuronal loss are two primary characteristic factors in PD. Thus, many researchers are interested in investigating the potential links between ATR exposure and PD.

Besides the effect of ATR on DA in the nervous system, researchers have also found its effects on other systems. Liu* et al.* demonstrated that ATR caused mitochondrial damage during apoptosis in grass carp [23]. Another study found that ATR induced apoptosis in splenocytes [24]. Other researchers found that ATR induced apoptosis in PC12 cells by altering the expression of p53, caspase-3, and caspase-9 [25]. Although ATR induced disruption of spontaneous locomotor activity and alteration of striatal dopaminergic systems has been reported in rats [26], few reports have explored the mechanisms underlying ATR induction of degenerative lesions in neuronal cells. Because there are several possible modes of cell death, including apoptosis, autophagy and necrosis, it is necessary to determine the mechanism by which ATR exposure induces dopaminergic neuron death. Based on previous investigations of the dopaminergic toxicity of ATR in rat striatal slices [27] and the proposed mechanisms involved in DA homeostasis [28], we hypothesized that ATR may lead to dopaminergic neuron degeneration via combined apoptosis- and autophagy-related pathways* in vivo*.

## 2. Results and Discussion

### 2.1. Results

#### 2.1.1. Body Weight and Physical Appearance

There were no obvious treatment-related changes in body weight or physical appearance during the three months of ATR treatment (data not shown).

#### 2.1.2. Effects of ATR on Histopathology

**Figure 1:**
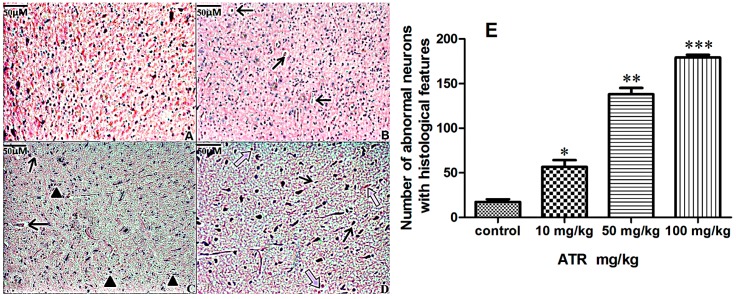
Histopathological changes in rat striatum treated with atrazine. Paraffin-embedded rat striatum sections were stained with hematoxylin and eosin as described in Materials and methods. (**A**) Normal histological structure (control group); (**B**) Atrazine group (10 mg/kg) shows nerve cell shrinkage (arrows); (**C**) Atrazine group (50 mg/kg) shows microglia infiltration (filled triangles) and neuron shrinkage (arrows); (**D**) Atrazine group (100 mg/kg) shows nerve cell shrinkage (arrows) and disappearance of Nissl body (hollow arrows). Scale bars = 50 µm; (**E**) Quantification of degenerative neurons. Data are presented as means ± SEM. * *p < *0.05, ** *p < *0.01, *** *p < *0.001,* vs.* the control group.

**Figure 2:**
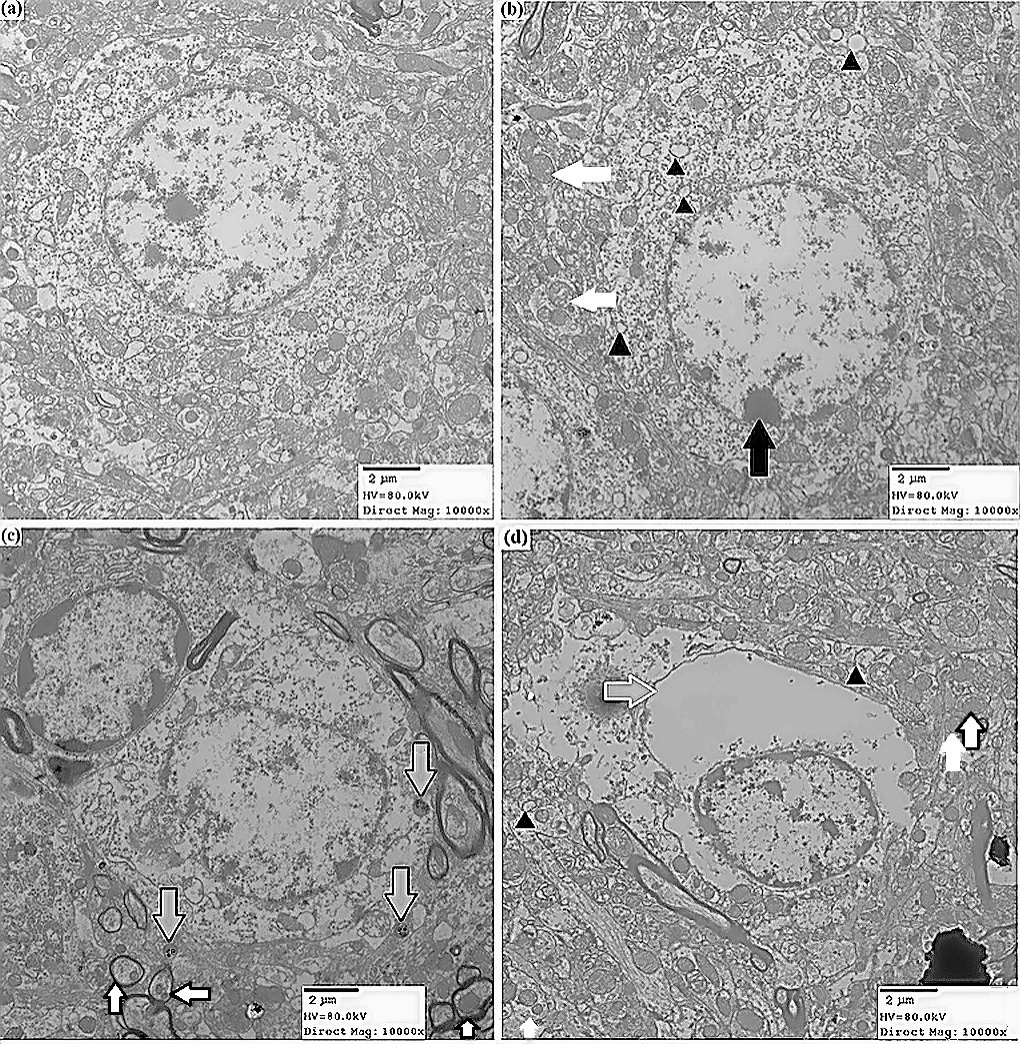
Ultrastructural properties of neuronal cells in rat striatum treated with atrazine. Representative ultrastructural images were taken with a transmission electron microscope. (**a**) The control group shows the normal ultrastructure of neuronal cells; (**b**) In the 10 mg/kg ATR group, neuronal cells exhibit chromatin margination (condensing into dense blocks (filled black arrow)), swelling and cristae fusion of mitochondria (filled white arrow), and mitochondrial vacuolization (filled black triangles); (**c**) The 50 mg/kg ATR group shows mitochondrial autophagy (black edge filled white arrow) and apoptotic bodies (black edge filled grey arrow); (**d**) The 100 mg/kg ATR group shows widened perinuclear cistern (white edge filled grey arrow), swelling and cristae fusion of mitochondria (filled white arrow), mitochondrial vacuolization (filled black triangles) and mitochondrial autophagosomes (black edge filled white arrow). Scale bars = 2 µm.

Histopathological examination of the striatum revealed degenerative changes after three months of ATR treatment, and the number of degenerative neurons increased with the increasing doses of ATR exposure (Figure 1). No degeneration was observed in the control group (Figure 1A,E). Sporadic neuronal pyknosis in the ventral midbrain was induced by 10 mg/kg ATR, and the number of degenerative neurons in 10 mg/kg ATR group slightly increased when compared with the control group (*p* < 0.05) (Figure 1B,E). Less neuronal pyknosis, Nissl body side shift (Figure 1C) and massive nerve cell shrinkage (Figure 1D) were observed in the 50 and 100 mg/kg ATR groups. The number of degenerative neurons in 50 mg/kg ATR group significantly increased (*p* < 0.01) and further increased in 100 mg/kg ATR group (*p* < 0.001) when compared with the control group (Figure 1E). Ultrastructural observations by transmission electron microscopy (TEM) showed perinuclear cistern widening, chromatin margination or disappearance, apoptotic chromatin condensation under the nuclear membrane-fixing ring, mitochondrial vacuolar degeneration and mitochondrial autophagy (a double membrane structure enclosing the mitochondria). All of these characteristics occurred in cells undergoing a retrogressive process (Figure 2).

#### 2.1.3. ATR Reduces TH-Positive Neurons in Rat Ventral Midbrain

**Figure 3:**
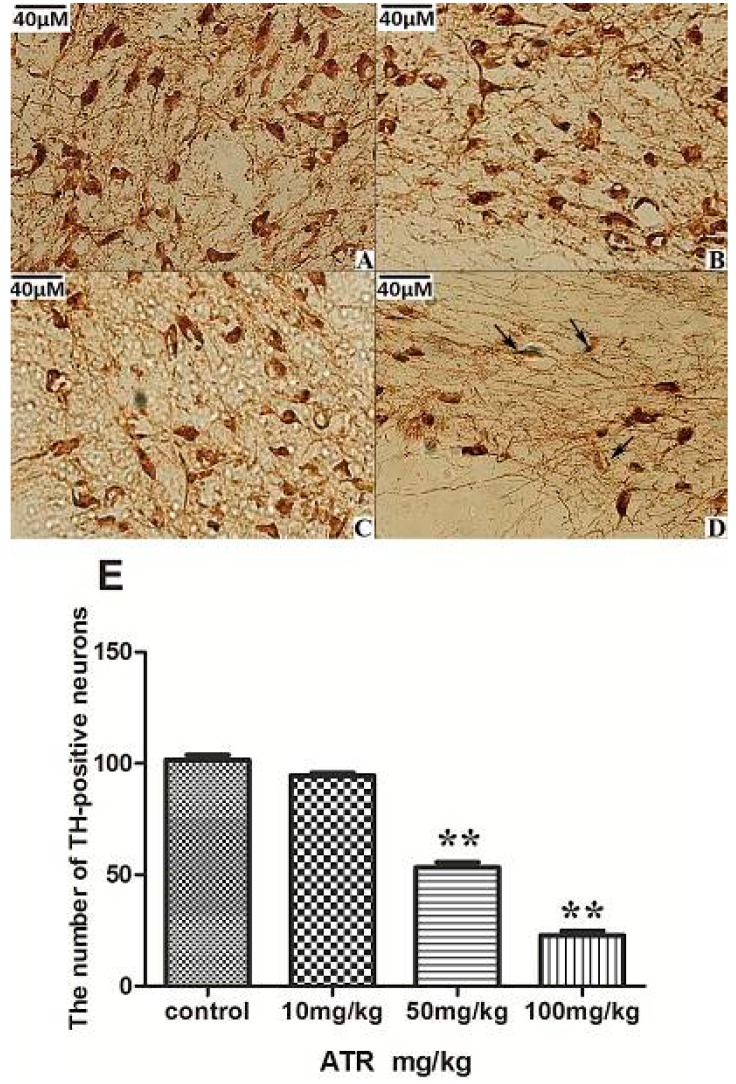
The effects of 90 days of ATR exposure on the number of TH-positive neurons in the ventral midbrain of rats. (**A**) Control group; (**B**) 10 mg/kg ATR group; (**C**) 50 mg/kg ATR group; (**D**) 100 mg/kg ATR group. Significant atrophy of TH-positive neurons (black arrow); (**E**) quantification of TH-positive neurons. Bars represent five animals per group. Data are presented as means ± SEM. ** p < 0.01,* vs.* the control group.

Immunohistochemical analysis showed a slightly, but not significantly, reduced number of TH-positive neurons in the 10mg/kg ATR group when compared with the control group (Figure 3A,B and E). The number of TH-positive neurons in the 50 mg/kg ATR group was significantly reduced when compared with the control group (*p* < 0.01), and mild cellular atrophy began to appear (Figure 3C,E). The number of TH-positive neurons was further reduced in the 100 mg/kg ATR group when compared with the control group (*p* < 0.01), and the cell bodies of those neurons exhibited significant atrophy (Figure 3D,E, black arrow).

#### 2.1.4. ATR Alters the mRNA Levels of Several Autophagy- and Apoptosis-Related Genes

**Figure 4:**
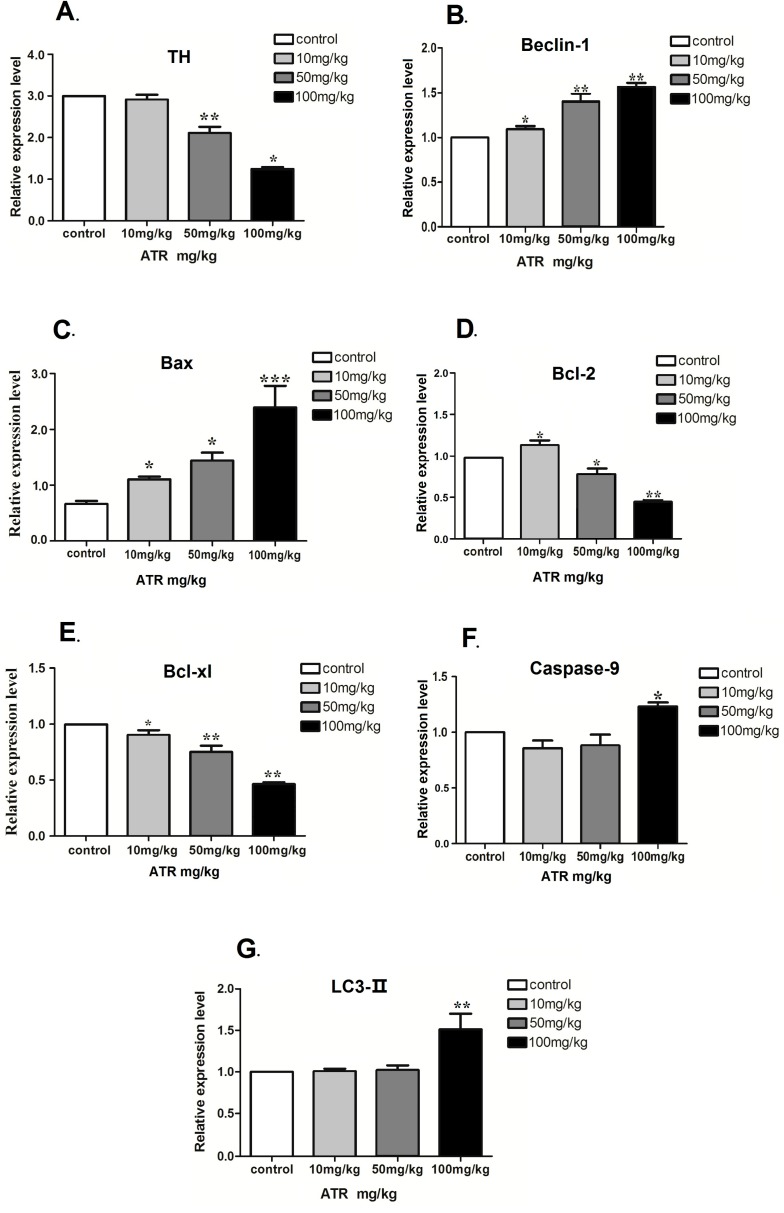
ATR induces changes in the mRNA expression of several autophagy and apoptosis related genes. The mRNA expression of TH (**A**); Beclin-1 (**B**); Bax (**C**); Bcl-2 (**D**); Bcl-xl (**E**); caspase-9 (**F**) and LC3-II (**G**) in the ventral midbrains of rats exposed to ATR for three months were measured by real-time PCR. Each group consisted of 10 rats. Data are presented as the mean percent of the control ± SEM. Expression levels were standardized to β-actin. * p < 0.05, ** p < 0.01, *** p < 0.001 when compared to the control group. A representative experiment is shown.

The changes in mRNA levels of Beclin-1, Bcl-xl, Bcl-2, Caspase-9, TH and LC3-II genes in ventral midbrains exposed to ATR for three months are shown in Figure 4. ATR exposure significantly decreased the mRNA expression of TH in 50 mg/kg and 100 mg/kg groups (*p *< 0.05; Figure 4A). ATR treatment significantly increased the mRNA expression of Beclin-1 in all treatment groups (Figure 4B). There was a significant dose-dependent increase in Bax mRNA expression in all treatment groups (*p* < 0.05; Figure 4C). Moreover, in conjunction with the increase in Beclin-1 and Bax, the change in Bcl-2 expression increased in the 10 mg/kg group, but decreased in the other treatment groups (*p* < 0.05; Figure 4D). There was a significant dose-dependent decrease in Bcl-xl mRNA expression in all treatment groups (*p* < 0.05; Figure 4E). Caspase-9 and LC3-II mRNA levels in the ventral midbrain were significantly increased in the high-dose group of 100 mg/kg ATR compared with the control group (*p* < 0.05; Figure 4F,G).

#### 2.1.5. Effect of ATR on the Amounts of Autophagy- and Apoptosis- Related Proteins

**Figure 5:**
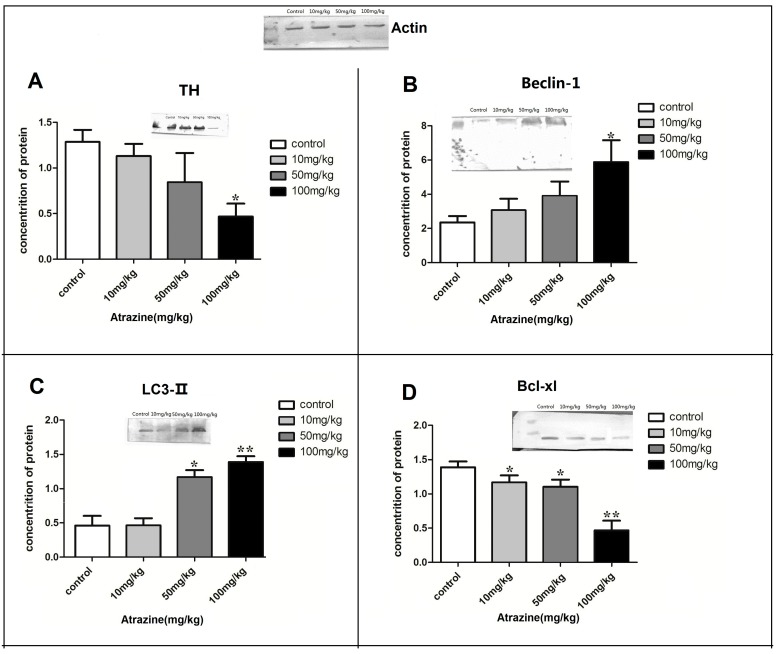

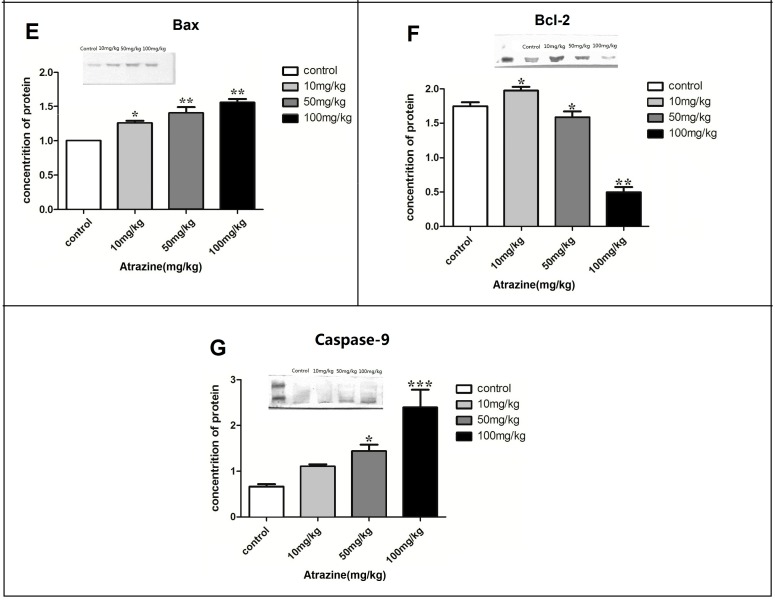
ATR alters the protein amounts of several autophagy- and apoptosis-related proteins. The amounts of Beclin-1 (**A**); Bcl-xl (**B**); Bcl-2 (**C**); Caspase-9 (**D**); Bax (**E**); TH (**F**) and LC3-II (**G**) proteins in the ventral midbrain were determined by Western blot analysis. Total pixel density was analyzed from a minimum of ten rats per group. Data are presented as mean percent of control ± SEM. * p < 0.05, ** p < 0.01, *** p < 0.001,* vs.* the control group.

TH protein was significantly decreased in the high-dose ATR group when compared to the control group (*p* < 0.05; Figure 5A). Beclin-1 protein was significantly increased in the high-dose ATR group when compared with the control group (*p* < 0.05; Figure 5B). The amount of LC3-II protein was significantly increased in the 50 mg/kg and 100 mg/kg groups when compared with the control group (*p* < 0.05; Figure 5C). The amount of Bcl-xl protein was significantly decreased in all treatment groups when compared with the control group (*p* < 0.05; Figure 5D). The amount of Bax protein was significantly increased in all treated groups when compared to the control group (*p* < 0.05; Figure 5E). Bcl-2 amount increased in the 10 mg/kg group and decreased in the 50 mg/kg and 100 mg/kg groups when compared with the control group (*p* < 0.05; Figure 5F). Caspase-9 amount increased in a significant and dose-dependent manner in the 50 mg/kg and 100 mg/kg groups when compared with the control group (*p* < 0.05; Figure 5G).

### 2.2. Discussion

Previous studies reported that ATR exposure reduced striatal dopamine secretion and disrupted spontaneous locomotor activity [29]. However, the mechanisms underlying ATR-induced cell death in dopaminergic neurons are not yet understood. In the present study, we attempted to evaluate the way in which ATR caused the death of dopaminergic neurons in rats. We found that ATR induced autophagy- and apoptosis-related neurodegenerative effects on the dopaminergic neurons of adult male Wistar rats.

Because ATR remains in the body for over 48 h, and due to inappropriate personal protective equipment and unintentional overspray, significant levels of ATR and its metabolites can be detected in farm workers from developing countries. Thus, it is possible that an accumulation of the chemical above the critical threshold causes long-term effects in workers after repeated exposure. The ATR dosage (10–100 mg/kg BW) we chose in this study was based on ATR levels established in previous studies [30,31,32].

After ATR treatment, we observed degenerative histopathological alterations in the HE stained striatum, including neural cell pyknosis, Nissl body side shift, microglia hyperplasia, and the number of degenerative neurons increased in a dose-dependent manner. Our results indirectly indicated that ATR exposure induced functional degradation of the rat striatum. This observation is supported by other studies [33,34]. As a part of the nigrostriatal dopaminergic system, the striatal dopaminergic neurons projected to the substantia nigra pars compacta dopaminergic cells, while the ventral midbrain dopamine neurons are almost all exclusively projected to the sensorimotor cortex and the ventral part of striatum. Thus, the ventral midbrain and the striatum form a control loop. When dopaminergic neurons are affected by autophagy and apoptosis-related degeneration in the nervous system, ventral midbrain and the striatum are undergoing similar changes [28]. The damage of ventral midbrain dopaminergic neurons can cause different compensatory responses in striatal DA receptor subtypes. After damage to the ventral midbrain DA neurons, DA neurons in the striatum were not only changed in quantity but also changed in the cell distribution [35,36]. Thus, we use the striatum to perform the pathological examination. Additionally, electron microscopy showed karyopyknosis, significant mitochondrial autophagy, microglia cell infiltration and degeneration of both mitochondria and vacuoles. These observations further supported the hypothesis that ATR induces significant functional and degenerative damage in neurons. Therefore, we attempted to determine the mechanisms that may be involved in the response of dopaminergic neuronal cells to ATR exposure.

TH provides instructions for synthesizing dopamine in dopaminergic neurons, which is important for the normal functioning of the nervous system. Multiple* in vitro* studies have reported that TH is not a major ATR target and that TH expression and activity are not significantly affected by ATR [21,27,37]. However, in our experiments, the number of TH-positive neurons decreased significantly in the ventral midbrain in response to increased ATR. This indicates that the neurotoxic effect of ATR was an asymptotic and cumulative process. Using real-time RT-PCR and Western blot assays, we found that the mRNA and protein levels of TH decreased significantly in the high-dose ATR group. These results indicated that ATR did not just affect the number of dopaminergic neurons, but that it also inhibited the mRNA and protein expression of TH. However, there is a possibility that the decreased TH expression may have been due in part to the death of TH-positive cells. This decrease may also potentially be attributed to gene interactions that occur during dopaminergic neuron death.

The primary aim of this study was to identify possible mechanisms underlying ATR-induced dopaminergic neuron degenerative damage at the subcellular level using real-time PCR and Western blot analysis. We focused on LC3-II, Beclin-1, Bcl-xl, Bcl-2 and Caspase-9, which play central roles in cellular autophagy and apoptosis in rats. We observed that ATR induced autophagy- and apoptosis-related neurodegenerative damage in the rat ventral midbrain. We demonstrated that ATR exposure increased LC3-II, Beclin-1 and Caspase-9 mRNA and protein levels, but reduced Bcl-xl and Bcl-2 mRNA and protein levels in the ventral midbrain in a dose-dependent manner. The increased LC3-II and Beclin-1 in the 50 and 100 mg/kg groups indicated that ATR might have a role in cerebral neuronal autophagy induction. It has been reported that numerous chemicals can induce apoptosis and/or autophagy; and morphologic features of both cell death mechanisms can be observed concurrently in the same cell. Recent advances have been provided to study the relationship between autophagy and apoptosis [38]. Gui* et al.* found that glyphosate can induced apoptosis- and autophagy-related cell death through adjusting the content of Beclin-1 [39]. Lead induces learning and memory injury through autophagy and apoptosis by regulating α-synuclein and tau hyperphosphorylation in hippocampus [40]. Methylmercury inhibits human neural stem cells through induction of caspase-dependent apoptosis and autophagy [41]. It is well-known that autophagy exhibits a largely cytoprotective role in physiologically relevant conditions. Autophagy may act as a cytoprotective mechanism favoring stress adaptation and can be mediated in many circumstances by negative modulation of apoptosis [42]. In specific conditions, autophagy may also promote or collaborate with apoptosis, and vice versa [43]. Although apoptosis and autophagy are thought to have extremely complex interrelationships, they can overlap at various signaling steps. While the mechanisms mediating the complex regulation of apoptosis and autophagy are not yet fully understood, several important upstream signaling molecules mediating this crosstalk include the interaction between Beclin-1 and Bcl-2/Bcl-xL, caspase-mediated cleavage of autophagy-related proteins, and autophagic degradation of caspases [44]. LC3-II and Beclin-1 are autophagy related genes that are involved in autophagosome formation. Notably, LC3-II has been used as a molecular marker for autophagic activity detection [45]. Because it is a Bcl-2-interacting protein, Beclin-1 is a key factor in autophagy regulation, and it is regulated by the Bcl-2 family proteins through its BH3 domain. Thus, Beclin-1 could be a switching factor that determines whether cells undergo autophagy or apoptosis [46]. Additionally, Caspase-9 expression was significantly increased as ATR dosage increased. The balance between autophagy and apoptotic genes and proteins was altered, and this may have led to caspase activation. Beclin-1 improves caspase-9 activity, thereby enhancing apoptosis. Caspase-9 is the most important initial factor in the caspase family, and initiates the cell death program by activating its downstream effector caspase in the mitochondrial pathway [47]. We found that the mRNA and protein levels of Bcl-2 and Bcl-xl were decreased in all three ATR groups. Beclin-1 regulates autophagy and apoptosis by increasing the expression caspase-3 and interacting with Bcl-2 and Bcl-xl [48,49,50]. When Bcl-2 is not interacting with Beclin-1, autophagy occurs [51]. However, caspase mediates cleavage of Beclin-1-dependent autophagy and enhances apoptosis [52,53]. Therefore, both Bcl-2 and caspase-9 could activate the release of Beclin-1 [54,55]. In summary, Beclin-1 is a key hub for cell apoptosis and autophagy crosstalk, by increasing activity of caspase-9 to induce apoptosis-related neurodegeneration, and by interacting with Bcl-2 and Bcl-xL to lead to cell autophagy-related neurodegenerative effects. Bax is considered a pro-apoptotic protein that disrupts the function of Bcl-2 protein, and further promotes caspase-9 activation and apoptosis. In our study, upregulation of Bax and downregulation of Bcl-2, Bcl-xl and caspase-9 indicate that ATR induced apoptosis-related neurodegenerative damage in nerve cells. These neurons exhibited the morphological characteristics of cells undergoing apoptosis and autophagy, such as mitochondrial autophagosomes and apoptotic cell chromatin condensation.

## 3. Experimental Section

### 3.1. Ethics Statement

All animals in this study were treated in accordance with the criteria outlined in the Guide for the Care and Use of Laboratory Animals prepared by the National Institutes of Health. All experiments were assessed and approved by the Medical Ethics Committee of Harbin Medical University (Harbin, China).

### 3.2. Chemicals and Reagents

2-chloro-4-ethylamino-6-isopropylamino-s-triazine (Atrazine, ATR, 98% purity) was purchased from Trustchem (Shanghai, China). Tyrosine hydroxylase (TH), Bcl-2, Bax and Caspase-9 antibodies were purchased from Immunoway (Newark, NJ, USA). Beclin-1, LC3-II and Bcl-xl polyclonal antibodies were purchased from Abcam (New Territories, Hong Kong, China). The alkaline phosphatase-conjugated secondary antibody was acquired from Sigma (St. Louis, MI, USA). The immunohistochemistry (IHC) kit was purchased from Abcam (Shanghai, China). Primers were synthesized by Generay Synthetic Biotechnology Company (Shanghai, China).

### 3.3. Animals and Treatments

One hundred eight-week-old male Wistar rats were purchased from Vital River Experimental Animal Technology (Beijing, China). All rats were allowed one week of acclimatization in the housing facility prior to the beginning of experimental protocols. Rats were divided into groups, with 25 rats per group, and kept under a 12-h inverted dark/light cycle (lights on at 20:00) at a constant temperature (23 ± 5 °C). Rats were given ATR mixed with cornstarch (3% starch solution) by oral gavage at doses of 10, 50 and 100 mg/kg body weight (BW) per day (five times per week) for three months. Rats given the same amount of a distilled starch solution were used as the control group. ATR dosages were selected according to the reported median lethal oral dose (LD_50_) of >672–3000 mg/kg in rats [56], and the lowest observed adverse effect level of 3.5 mg/kg/day. BW was recorded once a week. Twenty animals in each group were euthanized by chloral hydrate (10%, 0.5 mL/100 g) 12 h after the last ATR administration. Immediately following euthanasia, the entire brain was removed and placed in a Petri dish filled with ice-cold physiological saline. The meninges were removed, and the vasculature, striatum and ventral mesencephalon were quickly isolated and weighed. The tissues from ten rats in each group were soaked in the formaldehyde/paraformaldehyde for pathological examination, and the tissues from the other ten were snap frozen in liquid nitrogen. Five rats in each group were deeply anaesthetized by chloral hydrate (10%, 0.3 mL/100 g) and used for IHC observation.

### 3.4. Histopathological Evaluation

#### 3.4.1. Light Microscopy

After fixation with 10% buffered formalin, striatum were dehydrated, made transparent, wax-impregnated, and then embedded in paraffin. Serial sections (5 μm) were prepared and stained with Harris hematoxylin and eosin (HE) for histopathological evaluation using a light microscope. The number of degenerative neurons in the study in Figure 1 was manually counted by my colleagues who are blinded to experimental groups. We chose the central one-third area of each slice for counting. The selected area of each slice was marked with a pencil and three fields of that selected area were counted. In total, forty slices and eight fields of each group were counted.

#### 3.4.2. Transmission Electron Microscopy

Striatum tissue was cut into 1 mm^3^ sized cubes and fixed in 2% glutaraldehyde containing 1% fresh paraformaldehyde for 48 h. Samples were next fixed for 2 h in a 1% osmium tetroxide solution, dehydrated in graded ethanol, and embedded in araldite. Ultrathin sections were cut, stained with uranyl acetate and lead citrate, and observed using a transmission electron microscope (JEM-2100, Jeol Electron Inc., Tokyo, Japan).

#### 3.4.3. Immunohistochemical Assays and Neuronal Counting

After anesthetization, rats were perfused through the aorta with saline (4 °C, 150 mL) followed by paraformaldehyde (4 °C, 4%, 400 mL). Ventral midbrains were removed and fixed for 4 h in 4% paraformaldehyde at 4 °C. After fixation, the tissues were placed in a 20% sucrose solution overnight. The next day, brains were rinsed once with phosphate buffered saline and embedded in optimum cutting temperature (OCT) compound. Frozen coronal sections were cut at 8 µm using a cryostat. Each slice was added 400 µL of TH primary antibody (1:1000) and incubated overnight (4 °C) following the instruction of the immunohistochemistry staining kit (Abcam, Cambridge, MA, USA). Slices were washed with PBS for 5 min (5 times) and added secondary antibodies for 30 min on the next day. Every fifth slice (60 slices in total) was used for neuron manual counting and data were analyzed by statistics software.

#### 3.4.4. RNA Isolation and Reverse Transcription

Total RNA was extracted from ventral midbrain tissue homogenate using Trizol (Invitrogen, Life Technologies, Grand Island, NY, USA), trichloromethane (Tianjin, China), dimethyl carbinol (Jiangsu, China) and ethanol (Beijing, China) according to the manufacturer’s instructions. The yield of each mRNA sample was determined using a NanoDrop2000 UV-Vis Spectrophotometer and the A260 nm/280 nm ratio (Thermo Fisher Scientific Inc., Waltham, MA, USA). Reverse transcriptase polymerase chain reaction (RT-PCR) was performed using equal amounts of mRNA (1.5 μg) and PrimeScript^®^ RT reagent kits with gDNA Eraser (Takara, Shiga, Japan). The reverse transcription procedure was set up according to the manufacturer’s instructions.

#### 3.4.5. Quantitative Real-Time PCR

Full gene sequences were obtained from the National Center for Biotechnology Information (NCBI) Nucleotide Database (http://www.ncbi.nlm.nih.gov/genbank/). All primers used in this study were designed and synthesized by Generay Biotechnology (Shanghai, China) (Table 1). Briefly, 0.4 μL of each sense and antisense primer were mixed with 18.2 μL SYBR^®^ Premix Ex TaqTM^II^ (Takara) in a final total reaction volume of 20 μL. DNA melting analysis was performed using a programmed temperature ramp from 60 to 95 °C in 2 min to produce a dissociation curve, from which the melting temperature (T_m_) was calculated. β-actin was used as a housekeeping gene. The reaction conditions were as follow: 95 °C for 30 s; then 40 cycles of 95 °C for 5 s, 60 °C for 30 s; 72 °C for 30 s. Melting curve analyses were performed to verify amplification specificity. The relative mRNA expression of each gene was normalized to the internal β-actin control. T_m_ values were experimentally determined using thermal dissociation curves generated from the target primers using an ABI PRISM^®^ 7000HT Sequence Detection System (Applied Biosystems, Life Technologies, Foster City, CA, USA).

**Table 1:** Gene-specific real-time quantitative PCR primers.

Gene	Primer (5′→3′)	Product Size (bp)
β-actin	Forward: TGTTGGCATAGAGGTCTTTACGGReverse: TGGGTATGGAATCCTGTGGCA	90
Beclin-1	Forward: GATGGTGTCTCTCGCAGATTCReverse: CTGTGCATTCCTCACAGAGTG	138
Bcl-xl	Forward: GCCACCTACCTGAATGACCACCReverse: TGAGCCCAGCAGAACTACACCA	174
Bcl-2	Forward: GGCATCCCAGCCTCCGTTAT”Reverse: GTGTGTGGGGAGCGTCAATA	121
Caspase-9	Forward: TCTACAAGGCAAGCCCAAGCReverse: CGTCCATCTGGTCATCTATTCC	169
TH	Forward: GTTCATCDDACGGCGACAGAReverse: ATGCCATATCATCGTCAGTTCCAC	152
LC3-II	Forward: AGAGCGAGAGAGATGAAGACGGReverse: TTGCCTTGGTAGGGGCTTAACA	125

#### 3.4.6. Western Blotting

Total protein was extracted from tissues using tissue lysis buffer (Beyotime Biotechnology, Shanghai, China) containing PMSF (Amresco, Solon, OH, USA). After 2 h incubation on ice, the lysate was centrifuged at 12,000 rpm for 10 min at 4 °C. The supernatant was collected and stored at −80 °C.

Total protein from the ventral midbrain (30 μg) was separated by 10% or 12% SDS-PAGE and then transferred onto PVDF membranes (Bio-Rad Laboratories, Inc., Hercules, CA, USA). Membranes were blocked for 1 h at room temperature with 1% bovine serum albumin in TBST. Next, blots were incubated overnight at 4 °C with anti-LC3-II, -Beclin-1, -Bcl-xl, -Bcl-2, -Bax, -TH or anti-Caspase-9 antibodies. The next day, blots were incubated for 1 h with anti-rabbit alkaline phosphatase secondary antibodies. Immunoreactive bands were visualized using Western Blue^®^ Stabilized Substrate Alkaline Phosphatase (Promega, Madison, WI, USA). Bands were visualized using a chemiluminescence detection system, and analyzed using Quantity One software (Bio-Rad, Hercules, CA, USA).

#### 3.4.7. Statistical Analysis

Prior to analyses, we confirmed data normality and homogeneity of variances. Significant differences were determined by ANOVA using SPSS 17.0 Software (SPSS Inc., Chicago, IL, USA). If significant differences were found, pairwise comparisons using the Dunnett *t* test were performed to determine the treatments that were specifically different from the control. Data were expressed as means ± S.E.M. Each experiment was performed at least three times. The level of significance for all tests was set at *p* < 0.05.

## 4. Conclusions

This study provides evidence that ATR induces autophagy- and apoptosis-related neurodegenerative effects on dopaminergic neurons* in vivo*. The decrease in the number of TH-positive neurons suggests that ATR is a dopaminergic toxicant. Rats exposed to ATR showed upregulation of Bax, Beclin-1 and LC3-II, and downregulation of Bcl-xl and Bcl-2 in both mRNA and protein levels. Capase-9 was also increased following exposure to ATR. The molecular mechanisms involved in this process, however, remain unclear. Therefore, further research is needed to fully understand the deleterious effects of ATR on the neural system, the mechanisms involved, and behavioral changes that may follow longer ATR exposure time.
